# Redefining Health Care Data Interoperability: Empirical Exploration of Large Language Models in Information Exchange

**DOI:** 10.2196/56614

**Published:** 2024-05-31

**Authors:** Dukyong Yoon, Changho Han, Dong Won Kim, Songsoo Kim, SungA Bae, Jee An Ryu, Yujin Choi

**Affiliations:** 1 Department of Biomedical Systems Informatics Yonsei University College of Medicine Seoul Republic of Korea; 2 Institute for Innovation in Digital Healthcare (IIDH) Severance Hospital Seoul Republic of Korea; 3 Center for Digital Health Yongin Severance Hospital Yonsei University Health System Yongin Republic of Korea; 4 Department of Cardiology Yongin Severance Hospital Yonsei University College of Medicine Yongin Republic of Korea

**Keywords:** health care interoperability, large language models, medical data transformation, data standardization, text-based

## Abstract

**Background:**

Efficient data exchange and health care interoperability are impeded by medical records often being in nonstandardized or unstructured natural language format. Advanced language models, such as large language models (LLMs), may help overcome current challenges in information exchange.

**Objective:**

This study aims to evaluate the capability of LLMs in transforming and transferring health care data to support interoperability.

**Methods:**

Using data from the Medical Information Mart for Intensive Care III and UK Biobank, the study conducted 3 experiments. Experiment 1 assessed the accuracy of transforming structured laboratory results into unstructured format. Experiment 2 explored the conversion of diagnostic codes between the coding frameworks of the *ICD-9-CM* (*International Classification of Diseases, Ninth Revision, Clinical Modification*), and Systematized Nomenclature of Medicine Clinical Terms (SNOMED-CT) using a traditional mapping table and a text-based approach facilitated by the LLM ChatGPT. Experiment 3 focused on extracting targeted information from unstructured records that included comprehensive clinical information (discharge notes).

**Results:**

The text-based approach showed a high conversion accuracy in transforming laboratory results (experiment 1) and an enhanced consistency in diagnostic code conversion, particularly for frequently used diagnostic names, compared with the traditional mapping approach (experiment 2). In experiment 3, the LLM showed a positive predictive value of 87.2% in extracting generic drug names.

**Conclusions:**

This study highlighted the potential role of LLMs in significantly improving health care data interoperability, demonstrated by their high accuracy and efficiency in data transformation and exchange. The LLMs hold vast potential for enhancing medical data exchange without complex standardization for medical terms and data structure.

## Introduction

Efficient health care data exchange is essential in medicine, particularly in facilitating continuous care [1]. Such data exchange becomes crucial when a patient uses multiple health care facilities or receives concurrent care, significantly influencing accurate treatment strategies. The emergence of personalized health care, becoming a cornerstone of modern medicine, necessitates the use of personal health records. This shift complicates data exchange processes as it demands the integration of data from multiple health care institutions, thereby posing substantial challenges [2,3]. Additionally, health care is increasingly including patient-generated health data (PGHD) from a diverse range of devices, including wearable technology, given the heterogeneity of products from different vendors [4-6].

Globally, health care systems contend with varying medical record formats and disparate coding systems. In the globalized health care paradigm, the mobility of patients across international boundaries introduces an added layer of complexity. The necessity for efficiently leveraging consolidated information from multiple nations escalates as international collaborative research broadens [7,8]. The *International Classification of Diseases* (*ICD*) has served as a global standard for diagnostic nomenclature, whereas the Systematized Nomenclature of Medicine Clinical Terms (SNOMED-CT) presents a detailed, structured, and multiaxial medical terminology system, gaining adoption worldwide, including in the United States and Europe. Divergent drug coding systems also continue to exist between the United States and Europe, with the RxNorm system adopted in the United States and the ATC system used across Europe. These discrepancies underscore the urgent need for robust and effective health care data exchange pipelines.

Over the years, significant attempts have been made toward the standardization of health care data amid notable challenges and limitations. Protocols, such as Health Level Seven International and Fast Healthcare Interoperability Resources (FHIR), have been introduced to enhance data exchange between medical devices and electronic health records [9]. However, despite their use, these standards often meet with noncompliance or suboptimal implementation. Specifically, FHIR has received criticism for its inherent complexity, obstructing its widespread adoption [10]. Moreover, a key obstacle in the exchange of health care data lies in the initial state of medical records, many of which are not stored following a universal standard. This inconsistency creates a significant challenge even before leveraging exchange protocols like Health Level Seven International and FHIR, designed to facilitate data sharing. The presence of standards does not automatically solve the issue of initiating the exchange when the starting point involves aligning diverse data formats.

The Observational Health Data Sciences and Informatics initiative represents one of the most robust efforts toward data standardization for research purposes. This initiative has developed a common data model and promoted data standardization across various institutions in accordance with this format, significantly accelerating data analysis across institutions [8,11]. Nonetheless, the standardization process has its limitations. One is a notable risk of information loss from the original data during standardization [12,13]. Despite sustained global efforts to transition data into standardized formats, the inherent challenges of standardization inhibit complete conversion and representation of the finer details in the original data. Therefore, effective data standardization remains a pervasive challenge in health care data exchange.

To address the challenges associated with data standardization, we attempted to explore alternatives beyond traditional approaches. A potential solution might be a system that supports flexible communication of raw data, for example, in natural language, permitting the end user to process and interpret data as required, thereby reducing the necessity for strict standardization. Large language models (LLMs), such as ChatGPT, which are designed to produce contextually relevant and coherent natural language responses based on input data, might be promising tools in this regard. Leveraging the capabilities of LLMs can enhance natural human interaction and streamline the management and summarization of extensive language-based data sets. Multiple studies have reported these potential applications of LLMs in the medical field; for example, mining medical text data for relevant clinical information, summarizing patient records and research findings, inferring medical outcomes from complex case histories, and reviewing medical literature to identify trends and validate clinical practices [14-17]. Consequently, if LLMs can proficiently transcribe patient data into text format and the receiving end can efficiently structure the resultant text data, then the intricate stages of data standardization may become redundant. This paradigm shift could significantly alter health care data exchange, heralding a future of seamless and universal data interoperability.

This study tests the hypothesis that text-based conversion and integration of hospital data in different databases would be more effective than current methods. To prove this, we focused on 3 aspects: accuracy of numerical data transformation into text and back, fidelity of text-based transformation for semantic data using *ICD* codes (ie, *ICD-9-CM* [*International Classification of Diseases, Ninth Revision, Clinical Modification*]), and effectiveness of extracting specific information, such as intensive care unit (ICU) medication details, during the transfer of text-format data. This study aims to demonstrate the potential of natural language–based systems for future health care data exchange.

## Methods

### Ethical Considerations

This study was approved by the institutional review board of Yongin Severance Hospital (9-2023-0037), and conducted in accordance with the Declaration of Helsinki, and the requirement for written informed consent was waived due to its retrospective nature.

### Data Sources

This study used 2 comprehensive public health care data sets, namely, the UK Biobank and the Medical Information Mart for Intensive Care III (MIMIC-III). The UK Biobank serves as a notable national and international health resource, monitoring the lives of 500,000 voluntary participants aged between 40 and 69 years across the United Kingdom from 2006 to 2010. This resource aims to bolster the prevention, diagnosis, and treatment of a wide range of serious and life-threatening diseases. The data set includes genotypic and phenotypic data, covering medical, lifestyle, and environmental aspects. The UK Biobank contains structured data from diverse diagnostic tests, medical and family histories, and various physical measures. The MIMIC-III database, crafted by the Lab for Computational Physiology at MIT, is a broad, publicly available resource containing the deidentified health data of approximately 40,000 critical care patients [18]. This data set includes demographic information, vital signs, laboratory tests, and medications, among other features. It is valued for its over 2 million free-text clinical notes, presenting a rich source of natural language medical data.

This study used ChatGPT (version 3.5; OpenAI), an artificial intelligence model recognized for its exceptional performance among universally applicable models [19-21]. Given that our primary aim was to assess the ability of LLMs to facilitate health care data exchange in general scenarios, we opted against fine-tuning the model to prevent overspecialization to specific data sets. As a result, we used ChatGPT (version 3.5) in its original form, without any modifications. Furthermore, our focus was on testing the accuracy of information extraction and transformation rather than the creativity of the language model. Therefore, in all experiments, we set the temperature to 0 to ensure a deterministic output from the model.

**Figure 1:**
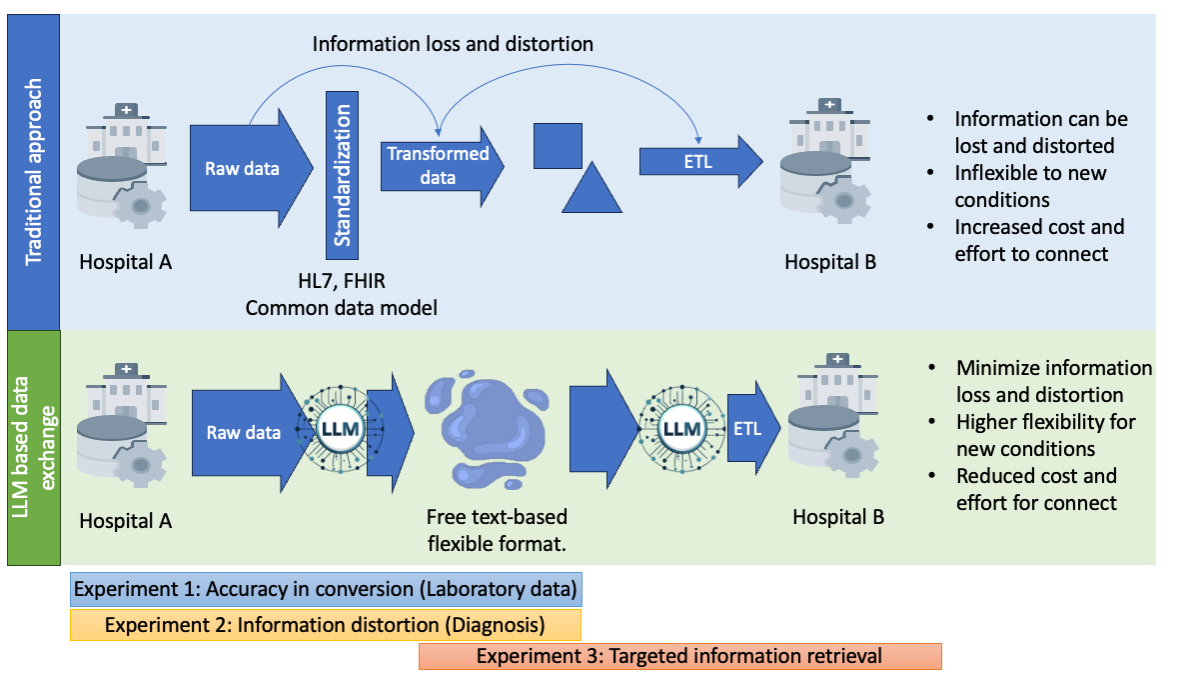
Comparison of traditional standardization-based and proposed text–based flexible data exchange processes. This figure illustrates the difference between the conventional process of data exchange, which relies on standardization, and our suggested method of flexible data exchange leveraging unstructured, text-based data. The traditional approach necessitates standardization, potentially leading to the loss or distortion of original information, diminished adaptability in new settings, and an increase in the cost and effort required for data exchange. Our proposed text-based, flexible data exchange process avoids these issues by reducing the loss of original information and boosting adaptability. This method is expected to cut down both the cost and effort involved in data exchange. At the bottom, we have delineated the 3 stages that our experiments aimed to validate. ETL: Extract, Transform, Load; FHIR: Fast Healthcare Interoperability Resources; HL7: Health Level Seven International; LLM: large language model.

The objectives of our study required the conduct of multiple trials featuring a range of prompts, a process termed prompt engineering. This process carries the potential risk of introducing an overfitting bias, which could boost the performance on specific data sets. Hence, we differentiated between the data used for prompt engineering experiments and those used to assess the performance of our experiments (Figure 1). Given the absence of a standardized methodology for prompt engineering, researchers often carry out this process manually, relying on trial-and-error approaches based on experience.

### Overview of the Experimental Design

**Figure 2:**
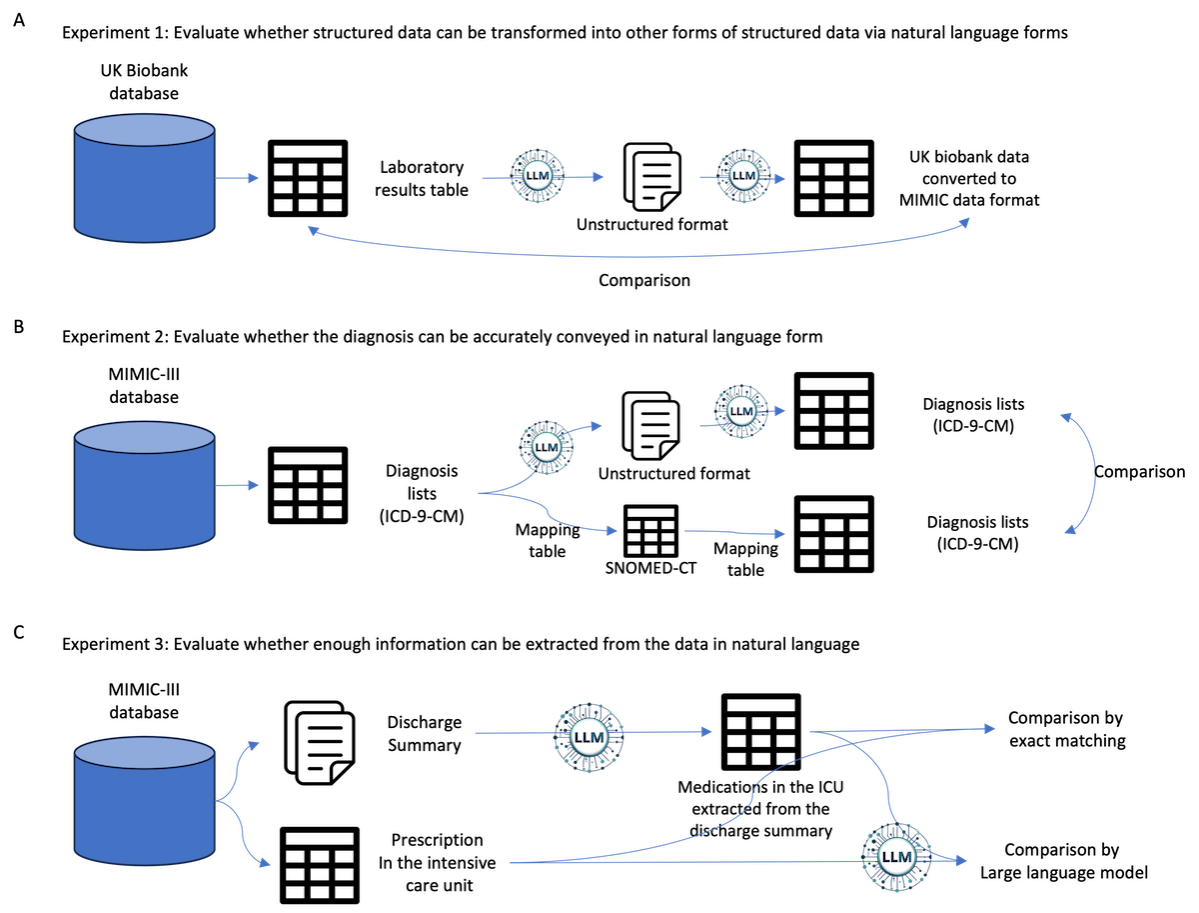
Overview of experimental approaches to evaluate LLM performance in data extraction and transformation. This figure outlines the methodologies used in experiments 1, 2, and 3. (A) In experiment 1, lab data from the UK Biobank data set was presented in natural language format and then restructured back into the MIMIC-III format using the LLM. The restructured data were then compared with the original laboratory data. (B) Experiment 2 evaluated the hypothesis that expressing diagnosis names in natural language might be more efficient than mapping them between varying coding systems. Diagnoses recorded in ICD-9-CM were rendered into natural language or SNOMED-CT, and then reverted into ICD-9-CM codes to examine the degree of information distortion. (C) In experiment 3, the LLM was assigned to extract targeted information concerning medications prescribed in the ICU from discharge summaries in the MIMIC-III database. The extracted information was then compared with the actual prescription records to assess the LLM’s accuracy in identifying and extracting details from unstructured text. ICD-9-CM: International Classification of Diseases, Ninth Revision, Clinical Modification; LLM: large language model; MIMIC-III: Medical Information Mart for Intensive Care III; SNOMED-CT: Systematized Nomenclature of Medicine Clinical Terms.

We hypothesized that converting a hospital’s data into text format and then integrating such data in another hospital’s database can be more accurate and comprehensive compared with other data transformation methods. To prove this, we tested 3 key aspects (Figure 2). First, we investigated whether the original data could be accurately conveyed when transformed into text (experiment 1). This involved converting numerical data into text and back into numerical form to check for any deviations from the original data. Second, we sought to validate that text-based transformation of information with numerical and semantic meaning would result in less distortion compared with rule-based transformations (experiment 2). To this end, we experimented with converting *ICD*-based diagnostic codes into text and back, comparing this with the results of converting them to and from the SNOMED-CT coding system. Finally, we evaluated whether the receiving institution could accurately extract specific desired information during the transmission of complex medical information in text form to another institution (experiment 3). In this experiment, we assumed that the content would resemble a discharge summary when all aspects of a patient’s hospital stay were compiled into a text format. Therefore, we aimed to test whether specific data, such as medication information prescribed in the ICU, could be accurately extracted from these summaries. In this experiment, we specifically worked under the assumption that the information to be extracted would be medication information prescribed in the ICU. From the 3 experiments, we aimed to evaluate the possibility of our hypothesis: a potential solution for health care data exchange in the future might be a system that supports flexible communication of raw data for example, in natural language (experiments 1 and 2), permitting the end user to process and interpret such data as required (experiment 3).

### Experiment 1: Evaluating Accuracy in Data Exchange via an LLM

To evaluate the feasibility of data exchange using an LLM, we randomly selected laboratory test result data from 1000 individuals from the UK Biobank data set. For each individual, we gathered laboratory test results and converted them into an unstructured format. Subsequently, the data were restructured to comply with the MIMIC-III data architecture. The prompts used throughout this process are detailed in Textbox 1.

**Summary of prompts used in experiments 1, 2, and 3.**

**Experiment 1**

Step 1: Translating laboratory test results into free text

“I have the following patient. Based on this information, summarize the patient’s condition in natural language. Make sure to include all the information presented. The values of the lab results should remain numerical. (For the Sex variable, 0 = female and 1 = male.)”

{List of lab results}

Step 2: Transforming free text data into the structured format

“I have the following patient.”

{Generated text from the above step}

“Extract and organize information on the following items.”

(Add the value next to the variable name with no further explanation.)

{Defined result extraction format}

**Experiment 2**

Step 1: Translating diagnosis codes to natural language text

“I have a diagnosis called {Diagnosis code}.

Describe it in natural language used by doctors and other health care professionals.

Write it as a single phrase of only a few words (less than 15 words but do not use abbreviations).

All semantics must be included.”

Step 2: Translating natural language text to diagnosis codes

“Where does {Descriptions on diagnosis} fit in the following categories?

{Categories according to International Classification of Diseases, Ninth Revision, Clinical Modification (ICD-9-CM)}

Provide the most appropriate ICD-9-CM code directly or choose one of the categories above.

Choose only one answer that seems the most relevant and answer in the following format.

The corresponding code: [Code (without periods): a description of the code].”

**Experiment 3**

Step 1: Extracting medication list from discharge summary

role: “system,” “content:” Your role is to interpret medical records.

role: “assistant,” “content:” I only need prescriptions from the ICU, not from the general ward or not from outside our hospital.

Organize by ingredient name, not generic name.

Never include medications on admission and discharge medications.

Exclude information before ICU admission or after ICU discharge, even if it is for a hospital stay.

In other words, exclude prescriptions that were written in a regular ward or emergency room.

Exclude any medications that may not have been prescribed in the ICU.

Finally, exclude all prescriptions for procedures, and tests. that are not prescriptions for medication.

role: “user,” “content:” Observing the following patient record, organize a list of medications prescribed during the ICU visit.

Organize them in the following format (Provide only the name, not the dose)

drug name 1

drug name 2

If any information on the medications prescribed in the ICU is unavailable, simply answer “None.”

Step 2: Converting drug names to ingredient names

{extracted drug list from the above step}

Organize the above medications by ingredient name.

If the drug is recorded by trade name, replace it with the ingredient name.

In the case of multiple ingredient names, record a representative one.

The format should be a single line of ingredient names with no further explanation, like this

List: Ingredient 1, Ingredient 2,...

Step 3: Comparing extracted drug information with actual prescription records

Here is the medication information extracted from the discharge summary.

{extracted drug list from the above step}

These are the medication details actually recorded in the prescription record.

{Ingredient list from the above step}

Organize the medication information extracted from the discharge summary by its actual inclusion in the prescription record.

Medications not mentioned in the discharge summary should not be listed.

The exact name of the medication may not be recorded, or a synonym may be used.

In these cases, mark the medication as actually prescribed.

For example, warfarin might be described as coumadin.

Record the same medication under different names as the one that was prescribed.

Match the same ingredient even if the added bases differ.

For example, the ingredient name of Lopressor is Metoprolol tartrate, but the ingredient must be confirmed as “true” even if it is Metoprolol.

Ingredient names may be written as abbreviations. For example, acetaminophen may be written as APAP.

Exclude P.R.N. prescriptions.

Exclude simple fluid prescriptions.

Provide only “true” or “false” information for each drug.

Do not provide Python code. Provide only the results in an array.

Fill in the blanks with a “true” or “false” result in the following format

{Defined result extraction format}

After the conversion to MIMIC-III data format via the LLM, we checked for potential omissions of information and any discrepancies in numerical values. We assessed the absence or presence of data omissions using sensitivity, specificity, and positive and negative predictive values. To assess the accuracy of the conversion, we used values transformed manually as the reference standard. Sensitivity indicated whether information from the original data set also existed in the transformed data. Conversely, specificity pertained to whether data absent in the original were also absent in the transformed data. The positive predictive value (PPV) referred to whether data present in the transformed data also existed in the original, whereas the negative predictive value determined whether data absent in the transformed data were also absent in the original. Numerical discrepancies were calculated only for test results presented in numerical format. They were assessed via the computation of the mean squared error between the original and transformed values.

### Experiment 2: Evaluating Possible Information Distortion During Conversion of Diagnosis Codes

In this experiment, we explored a scenario of diagnostic codes from the primary data set undergoing transformation for sharing across different institutions or to diverse end users. We aimed to clarify potential discrepancies emerging from transitions between the original and an alternate coding framework. Initially, we used a code-mapping table to facilitate the transition from one coding system to another. Subsequently, we reverted the transformed codes to the original coding framework, and then quantified discrepancies by comparing the reverted data against the primary data set. Using the MIMIC-III database, we converted diagnoses encoded in *ICD-9-CM* to SNOMED-CT, and subsequently reverted the same to *ICD-9-CM*. This conversion was based on a mapping table from a previous study [22]. Our proposed approach primarily leveraged the capabilities of the LLM, converting the primary coding structure into a natural text format. For a comparative analysis with the traditional approach, we recoded the text-converted diagnoses into the primary coding system (*ICD-9-CM*) using the LLM, as illustrated in Figure 2B. However, for this experiment, we excluded E and V codes (supplementary classifications for external causes of injury).

In assessing the accuracy of the restoration of diagnostic codes, we conducted evaluations based on the depth of the *ICD-9-CM* coding system. The highest level was labeled level 1, with each subsequent, more specific layer labeled level 2, level 3, and so forth. For instance, if the original data had been coded as “401.1 Hypertension, benign” but the restored data were denoted as “401.9 Hypertension, unspecified,” then the evaluation would be a mismatch at level 3. However, at level 2 granularity (ie, “401. Hypertension”), the codes were considered matching.

### Experiment 3: Assessing the Efficacy of LLMs in Extracting Targeted Information From Unstructured Medical Records

To evaluate the capability of our model in extracting targeted medical information from unstructured text, we selected narrative-style discharge summaries from the EVENTNOTES section of the MIMIC-III database, based on the assumption that they would reflect the comprehensive format typical of patient summaries transmitted between hospitals. These summaries provide a comprehensive account of a patient’s stay in the ICU, including clinicians’ assessments, patient medical history, laboratory results, interpretations of medical imaging, prescriptions, and ensuing care plans. This data set presents a detailed array of narrative insights that illustrate the complexities of patient care, diagnostics, and therapeutic strategies within the ICU context.

For this experiment, we specifically extracted discharge summaries documented by clinicians. These summaries encapsulated patient diagnoses, vital sign readings, current medication regimens, and other relevant status updates, all expressed in natural language. The prompts used in this process are presented in Textbox 1.

To evaluate the performance, we compared the information extracted from natural language with the information stored in structured tables. For this assessment, we made a random selection of 1000 discharge summaries, and we used structured data—prescription records—to verify the accuracy of the information retrieved through the LLM. Our focus was on assessing the PPV, representing the precision of the information extracted by the LLM. The extracted information was considered correct if it was also present within the structured data; otherwise, it was classified as incorrect. Notably, not all prescriptions are routinely documented in natural language by clinicians. Generally, only therapeutics significantly influencing the patient’s clinical status would be transcribed in the notes. As such, calculating the negative predictive value (ie, the number of medications not mentioned in the narrative notes that were actually not administered) was deemed impracticable. Similarly, sensitivity (ie, the degree to which prescribed medications are documented in narrative notes) and specificity (ie, the extent to which nonprescribed medications are not mentioned in narrative notes) could not be reliably estimated.

### Used Software

We accessed ChatGPT (version 3.5) via its API interface. We used Google BigQuery to manage and deploy the MIMIC-III and UK Biobank data sets. We used Python for certain tasks, such as assessing model performance.

## Results

### Features of the Extracted Data

In experiment 1, we used the lab test results of 1000 individuals randomly selected from the UK Biobank data set. For experiment 2, we used all diagnosis codes recorded within the MIMIC-III database. Finally, we used 1000 discharge summaries extracted randomly from the MIMIC-III database for experiment 3. Table 1 presents a detailed summary of the data used across all experiments.

**Table 1:** Summary of data used in each experiment.

	Experiment 1	Experiment 2	Experiment 3
Database	UK Biobank	MIMIC-III^a^	MIMIC-III
Data type	Laboratory test results	Diagnosis code (*ICD-9-CM*^b^)	Discharge summary
Number of records	502,396	651,047	59,652
Number of patients	502,396	46,520	41,127
Age (years), mean (SD)	56.53 (8.09)	64.43 (57.20)	58.35 (53.63)
Sex (male), n (%)	229,079 (45.6)	26,121 (56.2)	23,199 (56.4)
Length of text (number. of characters), mean (SD)	N/A^c^	N/A	9618.92 (5539.64)
Number of tests	11,973	N/A	N/A
Number of diagnosis codes	N/A	6984	N/A

^a^MIMIC-III: Medical Information Mart for Intensive Care III.

^b^ICD-9-CM: International Classification of Diseases, Ninth Revision, Clinical Modification.

^c^N/A: not applicable.

### Results of Experiment 1: Efficiency of the LLM in Data Transformation and Retrieval

In experiment 1, our objective was to assess the capability of the LLM in transforming and extracting laboratory results. We randomly selected the laboratory results of 1000 individuals from an initial data set of 502,396 individuals. This resulted in 11,996 data points spanning 13 distinct test items (excluding tests with null results). These data points were subsequently translated into natural language. Remarkably, only 23 items were lost during the transformation process, with 11,973 (99.8%) being successfully converted. Among the transformed data, 24 items did not match their original values perfectly. However, upon closer examination of these discrepancies, all inconsistencies were found to stem from the rounding off of decimal values. For instance, an original BMI value of 24.4383 was translated as 24.44. Consequently, the calculated mean squared error was a minimal 1.76e-07. Table 2 provides a comprehensive summary of errors for each laboratory test.

**Table 2:** Summary of experimental results from data transformation and extraction using LLM in experiment 1.

Variable	Raw data		After transformation		Number of data not transferred during the transformation process	Number of data with changed values during the transformation process	MSE^a^
	n, (%)	Mean (SD)	n, (%)	Mean (SD)			
Age	1000 (100)	56.94 (8.03)	1000 (100)	56.94 (8.03)	0	0	0
Sex	1000 (100)	0.47 (0.5)	1000 (100)	0.47 (0.5)	0	0	0
BMI	994 (100)	27.04 (4.78)	994 (100)	27.04 (4.78)	0	24	2.12×10^–6^
ALT^b^	919 (100)	23.55 (15.32)	919 (100)	23.55 (15.32)	0	0	0
AST^c^	918 (100)	26.13 (11.21)	918 (100)	26.13 (11.21)	0	0	0
Bilirubin	772 (100)	1.84 (0.81)	772 (100)	1.84 (0.81)	0	0	0
Creatinine	920 (100)	72.94 (18.65)	907 (98.6)	72.85 (18.69)	13	0	0
GGT^d^	920 (100)	39.06 (46.96)	920 (100)	39.06 (46.96)	0	0	0
HbA_1c_^e^	930 (100)	35.88 (5.66)	930 (100)	35.88 (5.66)	0	0	0
HDL^f^	846 (100)	1.46 (0.38)	846 (100)	1.46 (0.38)	0	0	0
LDL^g^	915 (100)	3.54 (0.88)	915 (100)	3.54 (0.88)	0	0	0
Platelet count	943 (100)	255.39 (59.79)	943 (100)	255.39 (59.79)	0	0	0
Triglycerides	919 (100)	1.73 (1.04)	909 (98.9)	1.74 (1.05)	10	0	0
Total	11996 (100)	42.9 (69.66)	11973 (99.8)	42.9 (69.71)	23	24	1.76×10^–7^

^a^MSE: mean squared error.

^b^ALT: alanine transaminase.

^c^AST: aspartate transaminase.

^d^GGT: gamma-glutamyl transferase.

^e^HbA_1c_: hemoglobin A_1c_.

^f^HDL: high-density lipoprotein.

^g^LDL: low-density lipoprotein.

### Results of Experiment 2: Analysis of Diagnostic Code Conversion (Mapping Table vs Text-Based Methods)

In the conversion, diagnostic codes were adapted based on a mapping table. Specifically, the original *ICD-9-CM* codes transitioned through SNOMED-CT before being remapped to *ICD-9-CM*. During this procedure, 5748 diagnostic codes expanded to 218,088 codes. This expansion may be attributed to the fact that specific mapping codes do not always allow for a direct 1:1 representation, leading to a 1:n relationship owing to challenges in semantic translation. As an illustration, the *ICD-9-CM* code for “Malignant pleural effusion: Malignant pleural effusion (51181)” was mapped as 2 distinct codes in SNOMED-CT: “Malignant pleural effusion (363346000)” and “Pleural effusion owing to malignant neoplastic disease (disorder) (860792009).” However, when converting through text, the mapping was nearly direct with a 1:1 ratio, ensuring that the 5748 original codes corresponded to 5748 records.

**Figure 3:**
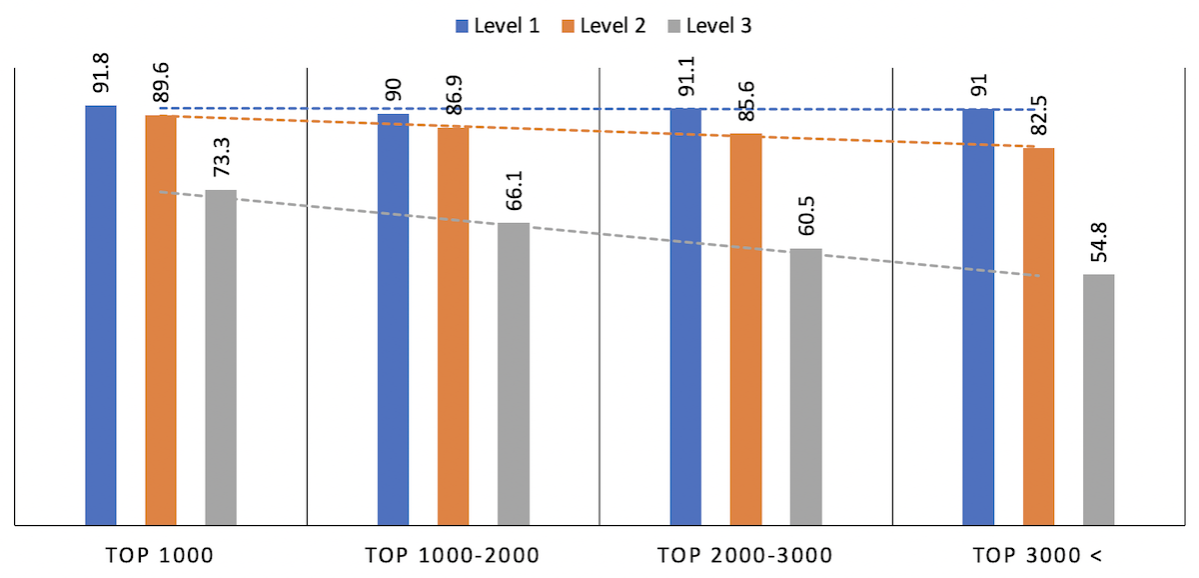
Results of converting diagnoses from ICD-9-CM (International Classification of Diseases, Ninth Revision, Clinical Modification) coding to natural language and back to ICD-9-CM. At the highest level, Level 1, most information aligned closely with the original data. However, accuracy decreased as the categories became more specific at levels 2 and 3. Notably, more frequently used diagnoses (toward the left on the x-axis) showed higher conversion accuracy.

Assessing the results before and after the conversion, we found that the mapping table achieved the following consistency values: 0.096 (21,000/218,088), 0.248 (54,068/218,088), and 0.626 (136,431/218,088) at levels 3, 2, and 1, respectively. Conversely, when relying on text-based methods, the consistency was higher, with corresponding values of 0.597 (3430/5748), 0.844 (4850/5,748), and 0.904 (5197/5748) for the same levels. An important observation pertained to the accuracy of conversion in relation to frequency use is that as the frequency increased, accuracy followed suit. Specifically, the top 1000 diagnostic names, based on their frequency, achieved values of 0.733, 0.896, and 0.918 at levels 3, 2, and 1, respectively, outperforming less common names. This observed relation was linear, as demonstrated in Figure 3. These results suggested that the frequent use of diagnostic names may provide better precision when shared between different databases.

During a review of the misclassified instances, we identified several cases as errors based on our evaluation standards. Notably, the semantic core of the original and converted phrases remained largely consistent. For example, we observed a transformation from “51881: Acute respiratory failure” to “78609: Respiratory abnorm NEC: Other respiratory abnormalities.” A comprehensive list of these misclassifications is provided in Table S1 in Multimedia Appendix 1.

### Results of Experiment 3: Effectiveness of the LLM in Extracting Relevant Information From Medical Records

In reviewing 1000 discharge summaries, the LLM identified a total of 5604 instances of medication prescriptions within the ICU setting. Of these, 2483 perfectly matched the entries in the prescription table, resulting in a PPV of 44.3%. When evaluated based on the shared active ingredient, we found a higher level of agreement, with 5055 out of the 5604 (90.2%) prescriptions showing alignment (Table 3). These findings, as exemplified by instances where “Acetaminophen” in the prescription information was referred to as “Paracetamol” in the discharge summaries and cases where “Metoprolol Tartrate” was simply documented as “Metoprolol,” underscore the tendency of physicians to note down familiar medication names. This behavior occurs instead of strictly adhering to the terminology prescribed in the prescription database. These examples highlight a preference for more universally recognized or familiar terms over the precise terminology listed in medical records. Despite this inherent variability in naming conventions, the LLM showed significant effectiveness in identifying and extracting the necessary information.

**Table 3:** Comparison of drug information extracted from natural language discharge summaries with prescription records.

	Number of medications, n (%)
Medications in the intensive care unit extracted from the discharge summary	5604 (100)
Medications that exactly matched the prescription name	2483 (44.3)
Medications semantically matched by large language model, including synonyms	5055 (90.2)^a^

^a^A total of 2572 medications were described using different terminology than the prescription.

## Discussion

Our research highlighted a new direction in health care by demonstrating the effective use of LLMs in medical data exchange. We aimed to overcome the current challenges related to data sharing among health care institutions, particularly owing to the unstructured nature of several medical records. We successfully validated all the key aspects we aimed to investigate, demonstrating the efficacy of our approach in enhancing health care data interoperability. The experiments revealed that converting hospital data into text format and subsequently integrating the converted data into another hospital’s database was not only feasible but also more accurate and comprehensive compared with traditional data transformation methods. Notably, our findings confirmed that the original data retained their accuracy and integrity when transformed into and back from the text format, a crucial factor in health care where precision is paramount. Moreover, our results indicated that text-based transformation, particularly for semantically rich information such as *ICD*-based diagnostic codes, resulted in significantly less distortion compared with rule-based methods. Finally, our method effectively enhanced medical data exchange by enabling precise extraction of specific information, such as ICU medication details, from text-transmitted data, thus, bolstering health care systems’ efficiency in integrating such data.

Our study highlights the significant role of LLMs in the field of health care informatics, demonstrating their transformative ability to manage, interpret, and share large volumes of medical data. Traditional data standardization methods, while important, have often been slow and challenging, creating barriers to fast and efficient data exchange. Our results showed that LLMs can not only interpret unstructured data but also convert it into easily understandable formats, greatly reducing the need for time-consuming standardization and allowing for faster data transfer.

Furthermore, the impact of our research extends beyond the clinical or institutional settings, affecting the broader area of personal health records. Integrating data from multiple providers into a single, unified record has always been a complex task. Different institutions often use varied formats, terminologies, and standards. Our work with LLMs suggested that these models can simplify this integration process. By understanding, transforming, and combining different data sources, LLMs can improve data sharing and enrich the information available.

LLMs’ adaptability in processing and interpreting structured and unstructured data hints at their potential to significantly enhance the handling of PGHD. Given the variety and unstructured nature of PGHD, from health diaries to wearable technology outputs, our findings suggest a promising avenue for applying LLMs to integrate and understand these diverse data sources more effectively. This capability aligns with our current results. Moreover, it opens up new pathways for creating more personalized and comprehensive approaches to patient care, leveraging the vast and untapped resources of PGHD.

Our study also provided significant insights into the process of converting diagnostic codes between standard coding systems, such as *ICD-9-CM* and SNOMED-CT. The higher number of diagnostic codes produced through this conversion process highlights the detailed and comprehensive nature of code capture enabled by the LLM. However, the approximate 1:1 ratio achieved in text-based conversions points to a more accurate and straightforward method. Importantly, these text-based conversions emphasize the major advantage of keeping the accuracy of the original data. For frequently used diagnostic terms, this method ensured that the core information from the original data remained consistent. Our examination of misclassifications revealed that, although identified as errors based on our criteria, several converted codes maintained similarity in their underlying meaning. Thus, despite “errors” in conversion, the core medical information is typically retained. Moreover, the direct relationship between the accuracy of conversion and frequency of diagnostic names hints at a possible inherent alignment of standard coding systems with commonly used terms. Our findings highlighted the critical importance of preserving data accuracy when moving between detailed medical coding systems. This aligns with the findings of previous studies, which suggest that using LLMs can lead to more accurate phenotype extraction from medical data [23,24].

Our findings have implications beyond individual health care systems and emphasize the potential for a significant change in the global health care landscape. Our data revealed that using LLMs can enhance international health information exchanges. Such improved communication can lead to better collaboration between countries, potentially benefiting patient care worldwide by ensuring that medical knowledge and practices are more consistently applied. Furthermore, our research points to a new direction in the design and operation of electronic medical record systems. The ability of LLMs to efficiently process and structure natural language data can make extracting, analyzing, and presenting medical data more straightforward. This not only allows for immediate analyses using the latest data but also promotes a more adaptable environment within electronic medical record systems to meet the dynamic needs of the health care sector, as illustrated in Figure 3.

While our study demonstrates the promising capabilities of LLMs in medical data processing, it is not without limitations. In this study, we used the GPT-3.5 model. Notably, using the newer GPT-4 might lead to better results, given that the efficiency of LLMs is continually improving. Comparative studies have demonstrated that GPT-4 performs better than its predecessors in various domains [25,26]. This progress in language model capabilities indicates the ongoing advancements we can expect. In addition to technological considerations, our reliance on specific data sets such as MIMIC-III and the UK Biobank, while providing valuable insights, introduces limitations regarding representativeness across diverse health care environments and languages. These data sets, representing particular health care settings and populations, may not fully encapsulate the complexity and diversity of global medical practices, especially in non–English speaking countries. This aspect underscores the necessity for broader research in applying LLMs across more varied data sets to ensure generalizability and applicability to different health care contexts. Regarding technological improvements, on-premise solutions can be expected to continue to improve in capabilities. Hence, our research serves as a foundation, showing the feasibility of data exchange based on LLMs. The accuracy and use of these transformations will be enhanced further in future versions. For institutions concerned with security implications, transitioning from externally provided models, such as ChatGPT, to an on-premise, self-built language model is a recommended strategy. Custom-built models can match the performance of GPT-3.5 for specific tasks [27,28]. Our choice to evaluate performance using the 3.5 version in this research provides a reference point and offers guidance for users considering the use of their custom language models.

Our research focused on specific data sets, and more extensive studies involving a wider range of data would be needed to confirm our initial observations. Moreover, the ability of LLMs to handle different types of unstructured data, each with its unique challenges, requires thorough assessment. Nevertheless, with ongoing advancements in artificial intelligence and machine learning, we expect that these challenges will be addressed, and the efficiency of LLMs in managing medical data will continue to improve. Future versions of LLMs, combined with careful validation, can bring significant improvements to health care informatics.

### Conclusions

In conclusion, our in-depth study provides important insights into the potential transformation of health care data exchange in the near future. The LLMs have a significant role in enhancing medical data sharing, ensuring both precision and efficiency. As technology advances and these language models become more refined, their role in health care data management and communication is anticipated to expand. Their potential goes beyond merely simplifying processes; they might also play a key role in minimizing errors, guaranteeing that medical professionals worldwide can access accurate and timely data. Ultimately, our findings suggest that with the incorporation of LLMs, the global health care landscape could become more unified, facilitating seamless knowledge transfer and collaboration among health care providers everywhere.

## Appendix

Multimedia Appendix 1 A comprehensive list of misclassifications in experiment 2.

## Abbreviations

FHIR: Fast Healthcare Interoperability Resources
ICD: International Classification of Diseases
ICD-9-CM: International Classification of Diseases, Ninth Revision, Clinical Modification
ICU: intensive care unit
LLM: large language model
MIMIC-III: Medical Information Mart for Intensive Care III
PGHD: patient-generated health data
PPV: positive predictive value
SNOMED-CT: Systematized Nomenclature of Medicine Clinical Terms

## Data Availability

The data sets analyzed during this study are available in the UK biobank [29] and MIMIC-III [30] repository.
